# Accessory Articulation of the Cervical Transverse Process: A Very Rare Anatomic Variant

**DOI:** 10.5334/jbsr.1348

**Published:** 2018-02-14

**Authors:** Stéphanie Braspenningx, Philip Simons

**Affiliations:** 1 Antwerp University Hospital and University of Antwerp, BE; 2 Onze-Lieve-Vrouwziekenhuis Aalst, BE

**Keywords:** Cervical spine, Transverse process, Anatomical variant, Computed tomography, Post-processing

## Abstract

We present a very unusual case of an accessory articulation of the transverse processes of C6 and C7. Only four previous cases have been described in English literature. Our case stresses the importance of computed tomography (CT) and post-processing images to discriminate this variant from posttraumatic or degenerative lesions. Multiplanar reformations and volume-rendered images should be added to the cervical spine CT protocol.

## Introduction

An accessory articulation of the cervical transverse process is an extremely rare anatomic variant caused by an elongated anterior tubercle of the transverse processes. Elongation of the anterior tubercle was first described by Lapayowker [1] in 1960. We found only four previous cases of an accessory articulation between these elongated anterior tubercles in the English literature [2,3,4,5], all of them at the C5–C6 level. We hereby report the first case ever described at the C6–C7 level. Post-processing images were able to demonstrate this rare variant more clearly than axial images alone, and make the differentiation from other conditions more straightforward.

## Case Report

A 55-year-old male patient with nuchal pain at C3–C4 level radiating to the left arm was referred for exclusion of a disc herniation. He underwent a cervical CT, not showing a disc herniation. However, it revealed a right-sided accessory articulation between the anterior transverse processes of C6 and C7. The transverse foramina of C6 and C7 showed a partial defect, respectively posterior and anterior. Clearly, the accessory articulation was an incidental finding, as being contralateral to the symptomatic side.

## Discussion

As already mentioned, an accessory articulation of the cervical transverse process is an extremely rare anatomic variant. The origin of the accessory articulation can be explained embryologically. Each vertebra is formed by three ossification centers: one so-called centrum and two neural arch centers. Transverse processes are formed by lateral extension of the neural arch centers. The costal portion of the transverse process is prominent in thoracic vertebrae, but fuses with the center of the transverse process in cervical vertebrae, the anterior tubercle being similar to thoracic ribs. However, when this costal portion is enlarged, it causes an elongation of the anterior tubercle of the transverse process, which is similar to a cervical rib.

**Figure 1:**
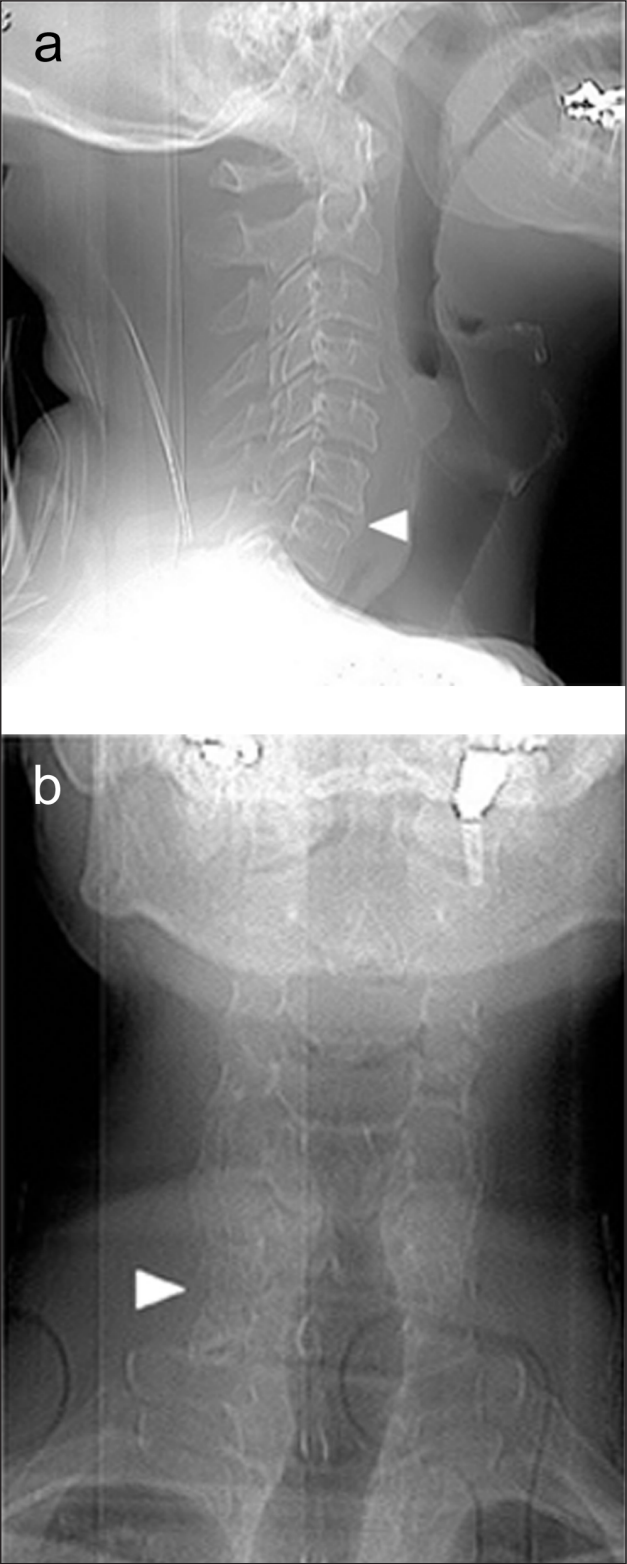
**(a)** Scout view showing an anomalous bony structure (arrowhead) between C6 and C7, arising from the transverse processes and projecting partially anterior to the vertebral bodies. **(b)** Scout view revealing a fine radiolucent line (arrowhead) between the right lateral masses of C6 and C7.

Conventional radiographs are often the initial imaging method for patients presenting with nuchal pain. In our patient, they were not obtained. However, retrospectively, one could already suspect an anomaly at the C6–C7 level looking at the lateral scout view (Figure 1a). There is an anomalous bony structure between C6 and C7 arising from the transverse processes and projecting partially anterior to the vertebral bodies. This excludes an ordinary osteophyte, as it would arise at the anterior border of the vertebral bodies rather than the transverse process. On the frontal images, a fine radiolucent line may be seen between the right lateral masses of C6 and C7 (Figure 1b).

**Figure 2:**
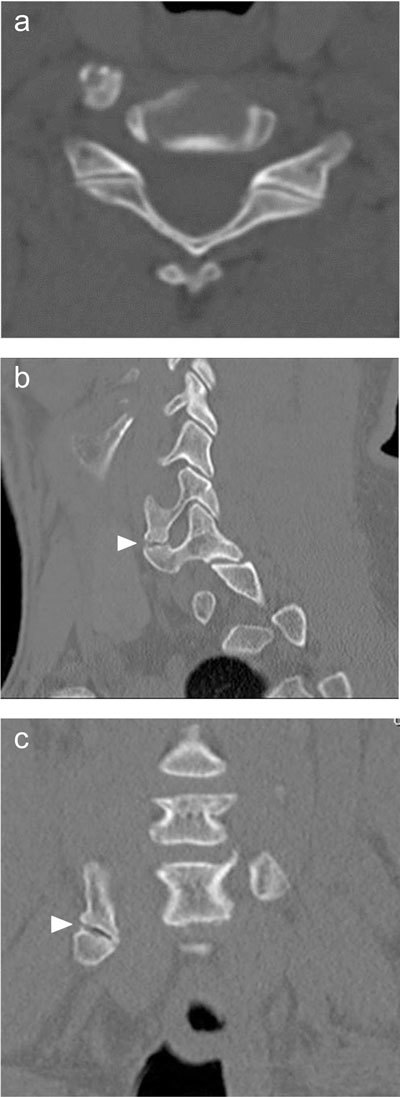
**(a–c)** Cervical CT with axial, sagittal and coronal reformations more clearly shows a right-sided elongation of the anterior tubercle of the transverse process of C6 and C7 and an accessory articulation between these anterior transverse processes (arrowhead).

**Figure 3:**
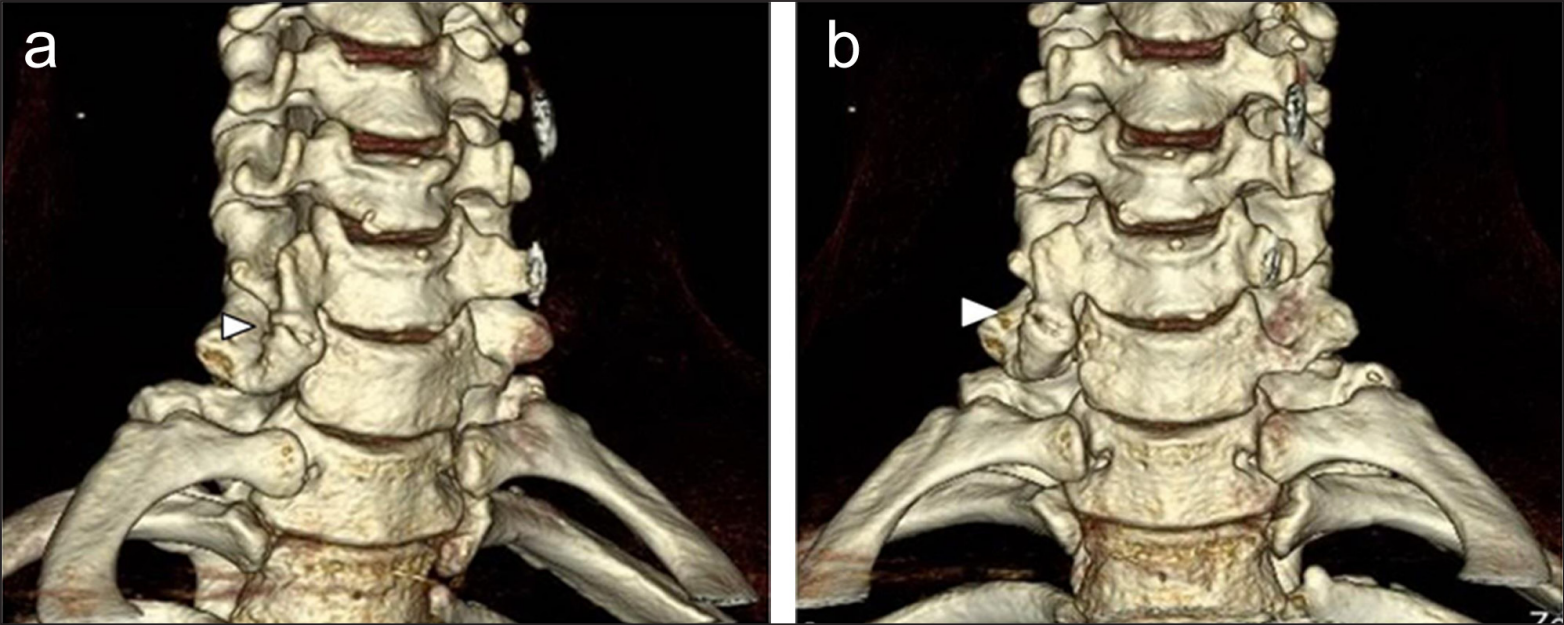
**(a–b)** Volume-rendered images clearly show the accessory articulation (arrowhead) as well as the complex osseous relations and may be helpful to detect possible areas of conflict.

CT better depicts the anomalous anatomy than conventional radiographs. Moreover, multiplanar reformations (Figure 2) and volume-rendered images (Figure 3) clearly show the complex osseous relations and give a better overview of possible areas of conflict than axial images alone. They should always be added to the examination protocol. The role of MRI is limited, but it may show early degeneration secondary to the accessory articulation and signs of nerve compression. Bone marrow edema may be demonstrated or ruled out in order to differentiate from possible fractures.

## Conclusion

In summary, we reported a very rare case of an accessory articulation of the transverse processes of C6–C7, and would like to stress the importance of CT and post-processing images to discriminate this variant from posttraumatic or degenerative lesions.
